# The Age, Sex, and Geographical Distribution of Self-Reported Asthma Triggers on Children With Asthma in China

**DOI:** 10.3389/fped.2021.689024

**Published:** 2021-09-03

**Authors:** Changhao Zhang, Yan Kong, Kunling Shen

**Affiliations:** Department of Respiratory Diseases, Beijing Children's Hospital, Capital Medical University, National Clinical Research Center of Respiratory Diseases, National Center for Children's Health, Beijing, China

**Keywords:** asthma trigger, asthma, children, age, sex, geographical distribution

## Abstract

**Background:** Asthma can be exacerbated by many triggers, and the heterogeneity of asthma triggers is clear among children with asthma. This study describes asthma triggers using a large-scale electronic dataset from the smartphone-based Chinese Children's Asthma Action Plan (CCAAP) app and aims to examine the difference in asthma triggers among different subgroups of children with asthma.

**Methods:** Data from the smartphone-based CCAAP app between February 22, 2017, and November 23, 2020, were reviewed, and children with asthma who reported their asthma triggers were enrolled. Eight common asthma triggers were listed in the software: upper respiratory infection (URI), allergen sensitization, exercise, emotional disturbances, pungent odors, air pollution/smog, weather change, and tobacco smoke. We compared the incidence of asthma triggers among different subgroups (<6 years vs. 6–17 years; boy vs. girl; eastern region vs. central region vs. western region).

**Results:** We enrolled 6,835 patients with self-reported asthma triggers. When compared by sex, boys had a higher proportion of exercise-triggered asthma than girls (boys vs. girls, 22.5 vs. 19.7%, *p* < 0.05). The proportion of patients <6 years of age with URI-triggered asthma was higher than that of patients 6–17 years of age (<6 vs. 6–17 years, 80.9 vs. 74.9%, *p* < 0.001). Patients 6–17 years of age were more likely than patients <6 years of age to report five of the asthma triggers: allergen sensitization (<6 vs. 6–17 years, 26.6 vs. 35.8%, *p* < 0.001), exercise (<6 vs. 6–17 years, 19.3 vs. 23.7%, *p* < 0.001), pungent odors (<6 vs. 6–17 years, 8.8 vs. 12.7%, *p* < 0.001), air pollution/smog (<6 vs. 6–17 years, 9.4 vs. 16.2%, *p* < 0.001), and tobacco smoke (<6 vs. 6–17 years, 3.5 vs. 5.3%, *p* < 0.001). In subgroups based on geographical distribution, asthma triggering of allergen sensitization was reported to be the most common in patients from the eastern region (eastern region vs. central region vs. western region, 35.0 vs. 24.6 vs. 28.0%, *p* < 0.001). Exercise-triggered asthma was found to be the most prevalent among patients from the central region (eastern region vs. central region vs. western region, 21.6 vs. 24.8 vs. 20.4%, *p* < 0.05). However, the proportion of patients with air pollution/smog as an asthma trigger was the lowest among those from the western region (eastern region vs. central region vs. western region, 14.1 vs. 14.1 vs. 10.8%, *p* < 0.05).

**Conclusion:** Children with asthma present different types of asthma triggers, both allergenic and nonallergenic. Age, sex, and geographical distribution affect specific asthma triggers. Preventive measures can be implemented based on a patient's specific asthma trigger.

## Background

More than 334 million people of all ages suffer from asthma worldwide (1). It is predicted that by 2025, the number of patients with asthma will increase by another 100 million (2). Asthma is the most frequent chronic disease in children. The goals of asthma management are to achieve good symptom control and to reduce the risk for asthma exacerbation (3). Asthma exacerbation not only seriously affects the quality of life of children with asthma but also places a great burden on families and society (4). Asthma triggers are the factors that lead to asthma exacerbations. To improve the control of asthma in children, it is necessary to analyze the characteristics of asthma triggers. A variety of asthma triggers have been found thus far, such as infections, exercise, allergens, environmental factors, and emotions (5). To further reduce asthma exacerbation, it is of great significance to understand the characteristics of asthma triggers and to formulate corresponding preventive measures (6, 7). Many factors impact asthma triggers, including age, sex, and geographical distribution differences.

A previous study conducted in the Kunming area in China reported impacts of age and sex on asthma triggers among children with asthma (8). However, no nationwide study of children with asthma in China focused on identifying factors correlating with asthma triggers. The objective of our study was to analyze the sex, age, and geographical distribution differences associated with asthma triggers among children with asthma in China. Although the geographical environment, ethnic characteristics, cultural background, and economic conditions may differ between China and other countries, our findings may still provide some reference for improving asthma management in children across different counties.

## Methods

### Study Design and Setting

To improve the management of children with asthma in China, the Chinese Children's Asthma Action Plan (CCAAP) was announced in Beijing in 2017. It was formulated by the China National Respiratory Diseases Clinical Medicine Research Center, the Respiratory Group of the Pediatric Branch of the Chinese Medical Association, and the Pediatric Professional Committee of the Chinese Medical Education Association. The CCAAP, which has a paper version and an electronic version, can help children with asthma cope with the loss of asthma control. The smartphone-based CCAAP app was used to help parents better achieve children's asthma management in their homes (9) and could be freely downloaded in China. This is a cross-sectional study based on smartphone-based CCAAP data.

Doctors from 31 provinces in China used the app for asthma management among children. Patients can maintain close contact with their doctors through the app and can send information to doctors through the app. In our study, 326 doctors maintained close contact with their patients. The doctors included pediatricians, pediatric respiratory physicians, and pediatric allergists. When the parents of the patients downloaded the app, they signed an informed consent form.

### Data Source and Extraction

This study analyzed data collected by the smartphone-based CCAAP app among children with asthma from February 22, 2017, to November 23, 2020. The patients' demographic characteristics (year of birth, sex, and geographical distributions) and other information (asthma triggers, history of inhalant allergen sensitization) were collected when the patients started to use the app. The inclusion criteria were as follows: (1) only questionnaires answered completely concerning age, sex, geographical distributions, and asthma triggers were considered appropriate for the study; (2) only children with asthma aged 0–17 years were included.

### Subgroups

Subgroup analyses of asthma triggers were conducted by age, sex, and geographical distribution. Age classification of patients was based on whether they were younger than 6 years old. All patients were divided into a <6-year group and a 6–17-year group. The sex subgrouping was based on boy and girl. According to the principle of combining the level of economic and technological development with geographical distributions in China, the geographical distribution subgroups consisted of the western region, the central region, and the eastern region.

### Perceived Asthma Triggers and Allergens

When patients registered in the app, they needed to report whether asthma attacks had previously been triggered by any of the following eight factors: upper respiratory infection (URI), allergen sensitization, exercise, emotional disturbances, pungent odors, air pollution/smog, weather change, and tobacco smoke.

Data regarding the history of inhalant allergen sensitization were collected. Specifically, inhalant allergens included mites, mold, cockroaches, pets, spring pollen, and autumn pollen.

### Statistics

Quantitative data are presented as the mean and SD. Frequency and percentage are used to represent categorical data. The chi-square test was to compare categorical clinical variables. *p* < 0.05 were considered statistically significant. Data were analyzed using SPSS 25.0 (SPSS Inc., USA).

## Results

### Baseline Characteristics

**Figure 1:**
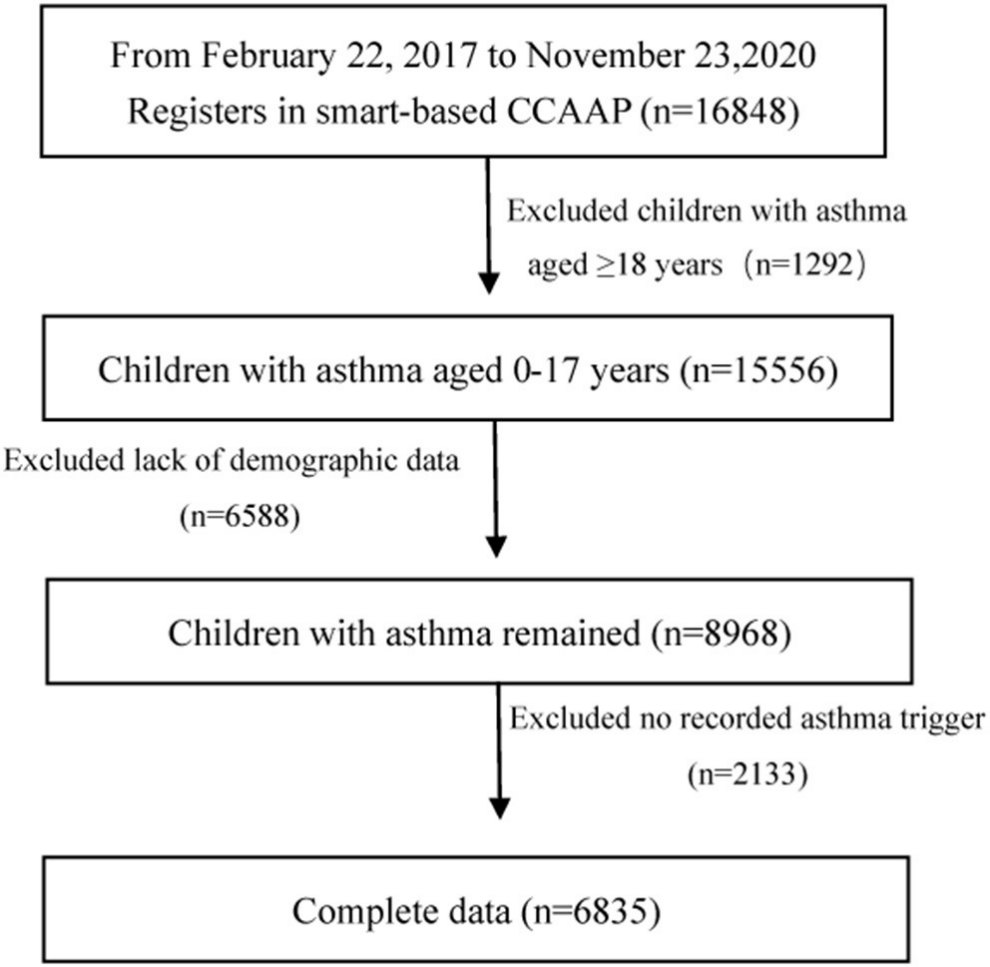
Study population flow chart (n, number; CCAAP, China Children's Asthma Action Plan).

From February 22, 2017, to November 23, 2020, 16,848 children with asthma used the smartphone-based CCAAP app. We excluded 1,292 patients aged ≥18 years. In addition, 8,721 patients with incomplete data on age, sex, geographical distribution, and asthma triggers were also excluded. In total, 6,835 patients were included. Figure 1 shows the patient screening procedure.

Table 1 provides the baseline characteristics of the patients. The mean age of all patients was 6.7 years. Boys comprised the majority of the patients (67.5%). Most patients were from the eastern region in China, accounting for 54.1% of the patient sample.

**Table 1:** Patient baseline characteristics.

**Factors**	***N* (%)**	**Mean ± SD (years)**
**Age**		
All participants	**6,835**	6.7 ± 3.2
<6 years	3,277 (47.9%)	4.2 ± 1.4
6–17 years	3,558 (52.1%)	9.1 ± 2.3
**Sex**	**6,835**	
Boy	4,613 (67.5%)	
Girl	2,222 (32.5%)	
**Geographical distribution ^#^**	**6,835**	
Eastern region	3,696 (54.1%)	
Central region	789 (11.5%)	
Western region	2350 (34.4%)	
**History of inhalant allergen sensitization**	**5,011** ^*^	
Dust mites	3,402 (67.9%)	
Mold	1,072 (21.4%)	
Cockroaches	286 (5.7%)	
Pets	746 (14.9%)	
Spring pollen	1,020 (20.4%)	
Autumn pollen	930 (18.6%)	
**The number of asthma triggers**	**6,835**	
One asthma trigger	3,143 (46.0%)	
Two asthma triggers	1,578 (23.1%)	
Three asthma triggers	1,212 (17.7%)	
Four asthma triggers	507 (7.4%)	
Five or more asthma triggers	395 (5.8%)	

* *The total number of participants is 6,835 among them 5,011 participants that had reported history of inhalant allergen sensitization*.

# * Eastern region: Fujian (n = 1119), Shandong (n = 630), Beijing (n = 575), Hebei (n = 401), Tianjin (n = 388), Liaoning (n = 273), Guangxi (n = 270), Guangdong (n = 98), Jiangsu (n = 78), Shanghai (n = 67), Zhejiang (n = 49), and Hainan (n = 18)*.

*Central region: Inner Mongolia (n = 244), Shaanxi (n = 178), Hunan (n = 145), Henan (n = 141), Jiangxi (n = 127), Anhui (n = 88), Hubei (n = 74), Heilongjiang (n = 26), and Jilin (n = 10)*.

*Western region: Shaanxi (n = 824), Chongqing (n = 231), Yunnan (n = 216), Guizhou (n = 198), Xinjiang (n = 153), Sichuan (n = 77), Gansu (n = 64), Qinghai (n = 39), Ningxia (n = 32), and Tibet (n = 2)*.

**Figure 2:**
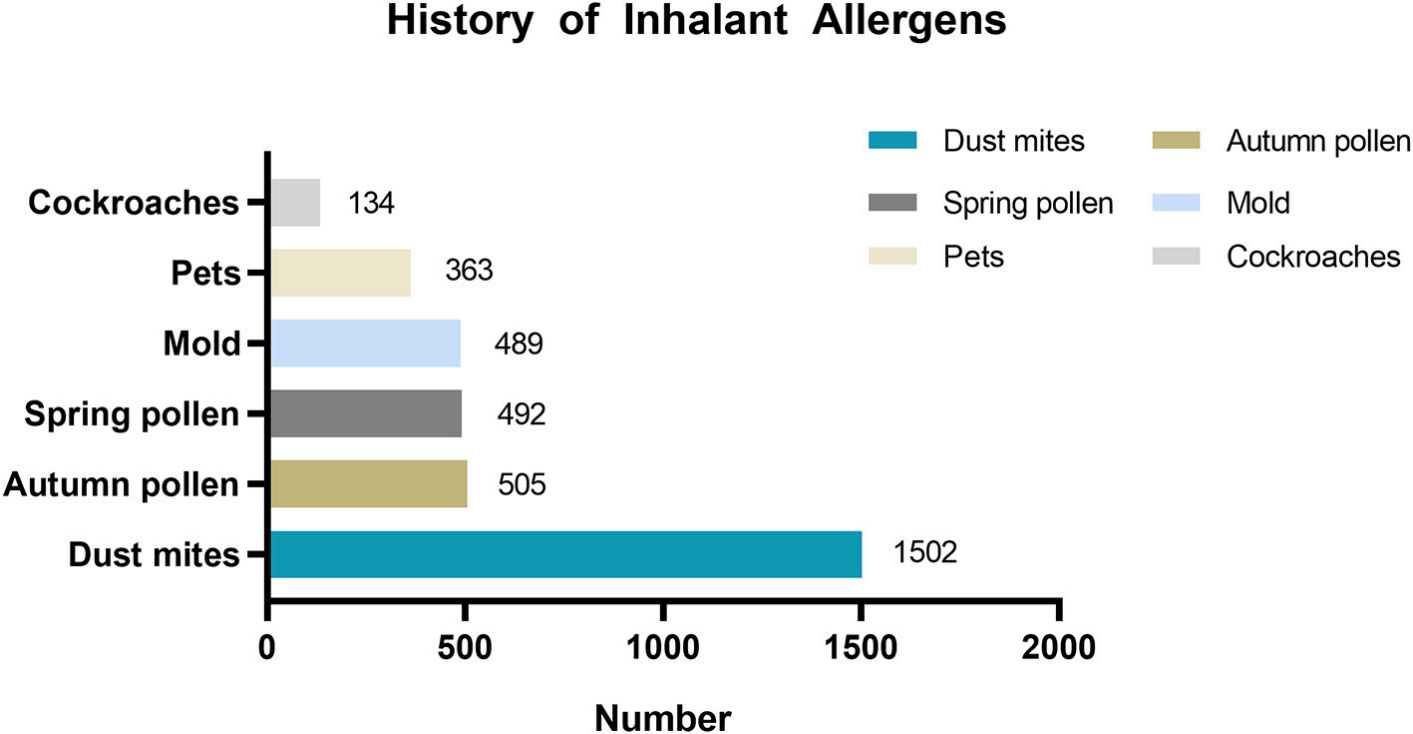
History of inhalant allergens sensitization distribution in patients with asthma trigger of allergen sensitization.

Table 2 shows the mean age of patients by asthma trigger. The mean age of children across all asthma triggers in the <6-year-old group was approximately 4 years old. In the 6–17-year-old group, the mean age of children with different asthma triggers was approximately 9 years old. There were 2,145 patients with the asthma trigger of allergen sensitization, and among them, 2,090 patients reported the type of inhalant allergen sensitization (Figure 2).

**Table 2:** Age distribution by asthma trigger.

**Factors**	***N* (%) (*N* = 6,835)**	** <6 years**	**6–17 years**	**Mean age (years)**
**URI**				
Yes	5,316 (77.8%)	4.2 ± 1.4	9.0 ± 2.3	6.6 ± 3.1
No	1,519 (22.2%)	4.2 ± 1.4	9.5 ± 2.5	7.3 ± 3.3
**Allergen sensitization**				
Yes	2,145 (31.4%)	4.4 ± 1.4	9.4 ± 2.4	7.3 ± 3.2
No	4,690 (68.6%)	4.1 ± 1.4	9.0 ± 2.3	6.5 ± 3.1
**Exercise**				
Yes	1,473 (21.6%)	4.5 ± 1.4	9.4 ± 2.5	7.3 ± 3.2
No	5,362 (78.4%)	4.1 ± 1.4	9.0 ± 2.3	6.6 ± 3.1
**Emotional disturbances**				
Yes	390 (5.7%)	4.4 ± 1.4	9.3 ± 2.6	6.8 ± 3.2
No	6,445 (94.3%)	4.2 ± 1.4	9.1 ± 2.3	6.7 ± 3.2
**Pungent odors**				
Yes	741 (10.8%)	4.4 ± 1.4	9.6 ± 2.6	7.5 ± 3.3
No	6,094 (89.2%)	4.2 ± 1.4	9.1 ± 2.3	6.7 ± 3.1
**Air pollution/smog**				
Yes	885 (12.9%)	4.7 ± 1.4	9.4 ± 2.4	7.8 ± 3.1
No	5,950 (87.1%)	4.1 ± 1.4	9.1 ± 2.3	6.6 ± 3.1
**Weather change**				
Yes	2,462 (36.0%)	4.3 ± 1.4	9.2 ± 2.3	6.9 ± 3.1
No	4,373 (64.0%)	4.1 ± 1.4	9.1 ± 2.4	6.7 ± 3.2
**Tobacco smoke**				
Yes	303 (4.4%)	4.3 ± 1.6	9.9 ± 2.6	7.8 ± 3.5
No	6,532 (95.6%)	4.2 ± 1.4	9.1 ± 2.3	6.7 ± 3.1

### Asthma Trigger Distribution Across Subgroups

**Figure 3:**
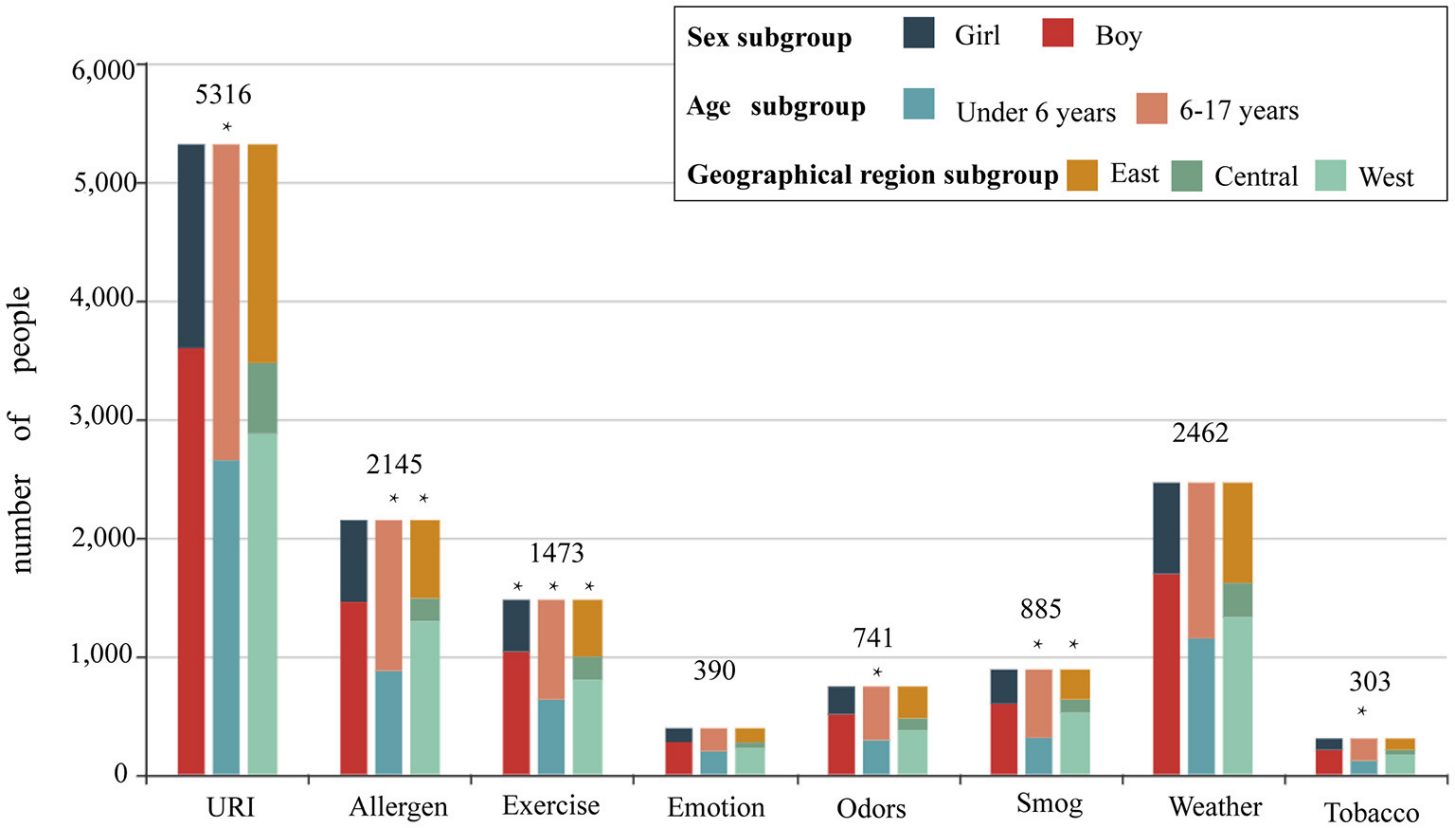
The proportion of different asthma triggers according to sex, age, and geographical distributions. Patients were divided into a boy group and a girl group according to sex; patients were divided into a <6-year-old group and a 6- to 17-year-old group according to age; and patients were divided into an eastern region group, a central region group, and a western region group according to geographical distributions.

Figure 3 and Table 3 describe the distributions of asthma triggers by subgroup. Patients with exercise-triggered asthma showed sex differences. The total percentage of patients reporting exercise-triggered asthma was 21.6%. Boys had a higher rate of exercise-triggered asthma when compared with girls (boys vs. girls, 22.5 vs. 19.7%, *p* < 0.05). However, no sex differences were found when examining other asthma triggers.

**Table 3:** The distribution of asthma triggers among subgroups.

	**Total number**	**URI**	**Allergen sensitization**	**Exercise**	**Emotional disturbances**	**Pungent odors**	**Air pollution/smog**	**Weather change**	**Tobacco smoke**
**Sex**									
Boy	4,613	3,597 (78.0%)	1,456 (31.6%)	1,036 (22.5%)	272 (5.9%)	508 (11.0%)	596 (12.9%)	1,692 (36.7%)	207 (4.5%)
Girl	2,222	1,719 (77.4%)	689 (31.0%)	437 (19.7%)	118 (5.3%)	233 (10.5%)	289 (13.0%)	770(34.7%)	96 (4.3%)
χ^2^		0.326	0.214	6.911	0.957	0.430	0.010	2.670	0.099
*p*-value		0.568	0.643	0.009*	0.328	0.512	0.921	0.102	0.754
**Age**									
<6 years	3,277	2,651 (80.9%)	873 (26.6%)	631 (19.3%)	195 (6.0%)	289 (8.8%)	308 (9.4%)	1,147 (35.0%)	115 (3.5%)
6–17 years	3,558	2,665 (74.9%)	1,272 (35.8%)	842 (23.7%)	195 (5.5%)	452 (12.7%)	577 (16.2%)	1,315 (37.0%)	188 (5.3%)
χ^2^		35.476	65.747	19.619	0.700	26.633	70.355	2.836	12.680
*p*-value		<0.001*	<0.001*	<0.001*	0.403	<0.001*	<0.001*	0.092	<0.001*
**Geographical distribution**									
Eastern region	3,696	2,874 (77.8%)	1,293 (35.0%)	798 (21.6%)	221 (6.0%)	374 (10.1%)	521 (14.1%)	1,326 (35.9%)	168 (4.5%)
Central region	789	597 (75.7%)	194 (24.6%)	196 (24.8%)	50 (6.3%)	99 (12.5%)	111 (14.1%)	290 (36.8%)	38 (4.8%)
Western region	2,350	1,845 (78.5%)	658 (28.0%)	479 (20.4%)	119 (5.1%)	268 (11.4%)	253 (10.8%)	846 (36.0%)	97 (4.1%)
χ^2^		2.768	51.660	6.953	2.899	5.141	15.129	0.219	0.901
*p*-value		0.251	<0.001*	0.031*	0.235	0.076	0.001*	0.896	0.637

* *p <0.05*.

The proportion of patients with asthma triggered by URI in the <6-year-old group was higher than that among patients in the 6- to 17-year-old group (80.9 vs. 74.9%, *p* < 0.001). For five asthma triggers, the percentage of patients reporting them was higher in the 6- to 17-year-old group than in the <6-year-old group: allergen sensitization (<6 vs. 6–17 years, 26.6 vs. 35.8%, *p* < 0.001), exercise (19.3 vs. 23.7%, *p* < 0.001), pungent odors (<6 vs. 6–17 years, 8.8 vs. 12.7%, *p* < 0.001), air pollution/smog (<6 vs. 6–17 years, 9.4% vs. 16.2%, *p* < 0.001), and tobacco smoke (<6 vs. 6–17 years, 3.5% vs. 5.3%, *p* < 0.001).

When examining geographical distribution, allergen sensitization was reported to be the most common asthma trigger in patients from the eastern region (eastern region vs. central region vs. western region, 35.0 vs. 24.6 vs. 28.0%, *p* < 0.001). Exercise as an asthma trigger was found to be the highest in patients from the central region (eastern region vs. central region vs. western region, 21.6 vs. 24.8 vs. 20.4%, *p* < 0.05). However, air pollution/smog as an asthma trigger was the lowest in the patients from the western region (eastern region vs. central region vs. western region, 14.1 vs. 14.1 vs. 10.8%, *p* < 0.05).

## Discussion

This study analyzed the distribution of several asthma triggers according to age, sex, and geographical distribution and found sex differences only in patients with exercise-triggered asthma. The proportion of patients with asthma triggered by URI was higher among patients <6 years old than among those 6–17 years old. By contrast, five other asthma triggers were more prevalent in the 6–17-year-old group than in the <6-year-old group: asthma triggers of allergen sensitization, exercise, pungent odors, air pollution/smog, and tobacco smoke. Allergen sensitization was reported more often in patients from the eastern region than in the other two regions. Exercise as an asthma trigger was reported more often in patients from the central region. Air pollution/smog as an asthma trigger was found less often among patients from the western region than among those in the other two regions. Based on the characteristics of different asthma triggers in children with asthma, corresponding prevention strategies are needed.

### URI

In the present study, URI was the most frequently reported asthma trigger among patients. This finding supports previous observations that asthma triggered by URI was prevalent in children with asthma (10). We also found that patients with URI as an asthma trigger were mainly localized in the <6-year-old group. Similar results were observed in the study of Jacobson et al. According to their report, respiratory infections were commonly found in preschool-age children and had negative impacts with asthma exacerbations (11). Viral infections are the leading cause of asthma exacerbation, especially in preschool-age children (12). A wide range of viruses can cause URI, with rhinovirus (RV) as the most commonly identified pathogen, particularly subtypes A and C (13). In addition, respiratory syncytial virus (RSV) and influenza virus are also common in daily medical practice. However, there is no safe and effective vaccine against RSV or RV at present. Currently, reducing asthma exacerbates, children with asthma can be recommended to receive influenza vaccines (14). In terms of drug treatment, palivizumab can be used for RSV prophylaxis. Multiple studies have shown that palivizumab can reduce the frequency of wheezing and the risk of asthma development (15–17). An animal experimental study showed that prophylactic azithromycin effectively reduced the severity of RSV infection (18). More clinical studies are needed to confirm this. Immunomodulators have a potential role in URI prevention, such as lysate and probiotics. At present, clinical trials have confirmed that OM-85 is effective and sufficiently safe for the prevention of respiratory tract infections (19, 20). More studies are needed to prove the benefits of immunomodulators and clarify their appropriate usage and dosages.

### Allergen Sensitization

Our results showed that the proportion of patients who reported asthma triggered by allergen sensitization was higher in the 6–17-year-old group than in the <6-year-old group. It is consistent with a study in Italy, Dondi et al. (21) found asthma exacerbation triggered by allergen sensitization mainly concentrated in children aged 6 or more. Darrow et al. (22) reported that pollen had a strong correlation with the increase in asthma and wheeze-related emergency department visits, especially in children aged 5–17 years old. We found that the proportion of patients who had reported asthma trigger of allergen sensitization in the eastern region was higher than that in the other two regions. Allergen sensitization may be related to the different geographic environments. Charpin et al. (23) found that the prevalence of House dust mites (HDM) allergen sensitization was related to the geographic environment. One of the strategies to prevent asthma exacerbations of children is to minimize the allergen in the environment. In addition to avoiding contact with allergens as much as possible, some indoor interventions, such as the use of air purifiers and the installation of ventilation systems, can also play an influential role (24).

### Exercise

In our study, a higher percentage of boys reported asthma triggered by exercise than girls, and the percentage was also higher in the 6–17-year-old group than in the <6-year-old group. A study in Japan found that age was associated with exercise-induced wheezing. They found a higher prevalence of exercise-induced wheezing among junior and high school pupils than primary school pupils (25). The study of Nigerian children with asthma reported that children with severe exercise-induced bronchoconstriction (EIB) were slightly older and more obese than children with non-severe EIB (26). In addition, our results showed that the proportion of patients who reported exercise-triggered asthma was the highest in the central region when compared to the other regions. This may be related to the cold and dry climate in the central region. Compared with warm, dry air, hyperventilation with cold, dry air produces a smaller increase in airway blood flow (27). Previous studies have shown that a higher absolute humidity (AH) of air is associated with a lower incidence of EIB after outdoor exercise tests in children (28). There are two potential preventive measures. First, good baseline control will reduce the incidence rate of EBI. Second, warming up before exercise training and using a short-acting Beta2 agonist (SABA) before exercise training can effectively avoid the exacerbation of asthma (29).

### Emotional Disturbances

We found that the percentage of patients with asthma triggered by emotional disturbances did not differ by sex, age, or geographical distribution. The results of previous studies differ from ours. There was a significant difference in the onset age of asthma symptoms between crying-induced bronchospasm (CIB) and non-CIB groups. Among patients with asthma symptoms, CIB children under 1 year old accounted for a larger proportion (30). Liangas et al. (31) reported that age was a significant determinant of mirth-triggered asthma, with the older age group showing a higher percentage of mirth-triggered asthma than the younger age group. Our research did not further classify emotional disturbances as crying or mirth. This may be the reason why our results are different from those of other studies. Severe psychological problems could increase the likelihood of asthma exacerbations (32, 33). Emotional fluctuations may come from the stress and pressure of childhood. Stress can increase airway inflammation and reduce the patients' response to short-acting bronchodilators and corticosteroids, resulting in poor asthma control (34, 35). In addition, emotional disturbances can also lead to other diseases manifesting as coughing and wheezing, such as tic cough, somatic cough syndrome, and cardiovascular disease. It is important to properly identify an asthma attack, especially during the COVID-19 pandemic. When observing children's psychological problems, parents should communicate in time, strive for professional help, relieve their pressure, and help children improve their emotion regulation ability.

### Pungent Odors

In our study, the proportion of patients who had asthma triggered by pungent odors was higher in the 6–17-year-old group than in the <6-year-old group. Previous studies have shown that patients with asthma report symptoms from exposure to odors and pungent chemicals (36, 37). A pungent odor can stimulate the trigeminal nerve and promote the release of neuropeptide mediators, which may trigger the asthma exacerbation. In addition, the influence of pungent odors on asthma attacks may be caused by cognitive mechanisms. Jaén et al. (38) found that the same odor was perceived as more irritating, when it was described as “harmful” than described as “healthful”. Children with asthma are generally afraid of inhaling pungent odors, which will adversely affect their health. In addition to avoiding exposure to pungent odors, patients also need to change their inherent perception.

### Weather Changes

Although the proportion of patients reporting weather changes as a trigger also did not differ by sex, age, or geographical location in our study, weather changes had effects on asthma attacks. Previous studies found that temperature changes and heavy precipitation were associated with asthma attacks (39, 40). A study in Shanghai found that regardless of the cold or warm season, high diurnal temperature differences, low relative humidity, and wind speed were all associated with a high risk of acute asthma exacerbation in children (41). Children with asthma and their parents should monitor the weather changes and avoid exposure to large temperature changes.

### Air Pollution/Smog

We found that the proportion of patients with air pollution/smog as an asthma trigger was higher in the 6–17-year-old group than in the <6-year-old group. Silverman et al. (42) found that PM2.5 and ozone were related to the increased risk of asthma hospitalizations. The estimated risks are age-dependent, with the strong associations appearing for the group age 6–18 years. In our study, the proportion of patients with air pollution/smog as an asthma trigger was the lowest in the western region when compared with the other two regions. Different areas have different levels of air pollution. Most cities in the western region are at high altitudes and are slightly polluted (43). In our study, we did not find the sex difference among patients with air pollution/smog as an asthma trigger. A multicity study in China has similar results, which reported that elevated concentrations of PM10 and NO_2_ were associated with increased hospital outpatient visits for asthma (44). Strickland et al. (45) found that air pollution was related to wheezing. In the daily management of asthma, environmental factors need to be considered, especially for children with severe or poorly controlled asthma. Clinicians should recommend patients to minimize outdoor activities during periods of poor air quality (46).

### Tobacco Smoke

In our study, the proportion of patients with tobacco smoke-triggered asthma was higher in the 6-17-year-old group than in the <6-year-old group. Children 6–17 years of age are affected not only by family members' smoking at home but also by more opportunities to be exposed to secondhand smoke in public places. Some older children may even actively smoke. A previous study has reported similar results with ours. Children between 1 and 6 years old were less affected by passive smoking. It was clear that the longer the duration of passive smoking was, the greater the impact on lung function (47). In our study, we did not observe a difference by sex in the proportion of patients with asthma triggered by tobacco smoke. However, Dong et al. (48) found an association between asthma-related symptoms and environmental tobacco smoke (ETS) exposure in boys but not in girls. Boys may be more susceptible to ETS than girls. ETS was the prevalent respiratory irritant, including nicotine, carbon monoxide, benzene, formaldehyde, and acrolein. When exposed to ETS, the prevalence of wheezing has been reported to increase (49). Wang et al. (50) found that ETS exposure was also associated with severe asthma attacks. Even under low-level exposure to ETS, children with asthma continue to show poor asthma control (51). Children with asthma and their families need to be aware of the hazards of ETS and understand that there is no safe level of ETS exposure. A tobacco-free environment is beneficial for children with asthma and requires the joint efforts of families and society.

### Advantages and Disadvantages

This article is the first large-scale multicenter study of asthma triggers in children with asthma based on CCAAP application data. This study will be the foundation for the related follow-up clinical research. It is of great significance to fully understand asthma triggers to support better management of children with asthma. In order to better prevent asthma attacks in children, targeted measures of different ages, sex, and geographical distributions should be implemented for asthma triggers.

Asthma attack data from self-reports are not as accurate as those recorded at the emergency department. We analyzed data about asthma triggers reported by parents/participants when registering the CCAAP application. However, we did not analyze the specific asthma triggers for each time an asthma attack was recorded in the app. We will study this point in depth in the future. In addition, this study analyzed each asthma trigger independently but did not further analyze the interactions between them. Instead, we focused on differences by age, sex, and geographical distribution for each asthma trigger. Finally, we did not record the specific types of allergens associated with each asthma exacerbation for patients with asthma trigger of allergen sensitization. However, as a supplement, we analyzed the history of inhalation allergens. The following research will further analyze the relationship between symptoms and asthma triggers to supplement this study field.

## Conclusion

Children with asthma reported different asthma triggers, both allergenic and nonallergenic. Age, sex, and geographical distribution affected specific asthma triggers. Preventive measures can be taken to avoid asthma attacks based on the specific asthma trigger.

## Data Availability Statement

The original contributions presented in the study are included in the article/supplementary material, further inquiries can be directed to the corresponding author.

## Ethics Statement

The studies involving human participants were reviewed and approved by Beijing Children's Hospital Ethics Committee. When the parents of the patients downloaded the app, they signed an informed consent form.

## Author Contributions

CZ drafted the initial manuscript, collected data, and carried out initial analyses. YK designed the data collection instruments and coordinated and supervised data collection. KS conceptualized and designed the study, and reviewed and revised the manuscript. All authors approved the final version of the manuscript as submitted.

## Conflict of Interest

The authors declare that the research was conducted in the absence of any commercial or financial relationships that could be construed as a potential conflict of interest.

## Publisher's Note

All claims expressed in this article are solely those of the authors and do not necessarily represent those of their affiliated organizations, or those of the publisher, the editors and the reviewers. Any product that may be evaluated in this article, or claim that may be made by its manufacturer, is not guaranteed or endorsed by the publisher.

## Abbreviations

AAP: asthma action plan
CCAAP: Chinese Children's Asthma Action Plan
URI: upper respiratory infection
RV: rhinovirus
PCV13: 13-valent pneumococcal conjugate vaccine
PPV23: pneumococcal polysaccharide 23-valent vaccine
EIB: exercise-induced bronchoconstriction
AH: absolute humidity
SABA: short-acting Beta2 agonist
ETS: environmental tobacco smoke.

## References

[1] Global Asthma Network. The Global Asthma Report. (2014). Available online at: http://globalasthmareport.org/2014/Global_Asthma_Report_2014.pdf.

[2] Bousquet J Bousquet PJ Godard P Daures JP. The public health implications of asthma. Bull World Health Organ. (2005) 83:548–54. 10.1590/S0042-96862005000700016 16175830 PMC2626301

[3] Global Initiative for Asthma Executive Committee. Global Strategy for Asthma Management and Prevention. (2021). Available online at: https://ginasthma.org/wp-content/uploads/2021/05/GINA-Main-Report-2021-V2-WMS.pdf.

[4] Kansen HM Le TM Meijer Y Uiterwaal CSPM Knulst AC van der Ent CK. Perceived triggers of asthma impair quality of life in children with asthma. Clin Exp Allergy. (2019) 49:980–9. 10.1111/cea.13407 31038823 PMC6851977

[5] Gautier C Charpin D. Environmental triggers and avoidance in the management of asthma. J Asthma Allergy. (2017) 10:47–56. 10.2147/JAA.S121276 28331347 PMC5349698

[6] Castillo JR Peters SP Busse WW. Asthma Exacerbations: Pathogenesis, prevention, and Treatment. J Allergy Clin Immunol Pract. (2017) 5:918–27. 10.1016/j.jaip.2017.05.001 28689842 PMC5950727

[7] Ramsahai JM Hansbro PM Wark PAB. Mechanisms and management of asthma exacerbations. Am J Respir Crit Care Med. (2019) 199:423–32. 10.1164/rccm.201810-1931CI 30562041

[8] Qi ZY Duan J Zhang Q Cao ZL Dai M Xiong JJ. Epidemiological survey of childhood asthma in Kunming City, China. Chin. J. Contemp. Pediatr. (2014) 16:910–3. 10.3969/j.issn.1003-4706.2016.08.009 25229958

[9] Zhu K Xiang L Shen K. Efficacy of Chinese Children's Asthma Action Plan in the management of children with asthma. Allergy Asthma Proc. (2020) 41:e3–e10. 10.2500/aap.2020.41.190010 31888788

[10] Xu D Wang Y Chen Z Li S Cheng Y Zhang L. Prevalence and risk factors for asthma among children aged 0-14 years in Hangzhou: a cross-sectional survey. Respir Res. (2016) 17:122. 10.1186/s12931-016-0439-z 27677381 PMC5039889

[11] Jacobson JS Goldstein IF Canfield SM Ashby-Thompson M Husain SA Chew GL. Early respiratory infections and asthma among New York City Head Start children. J Asthma. (2008) 45:301–8. 10.1080/02770900801911186 18446594

[12] Moser S Peroni DG Comberiati P Piacentini GL. Asthma and viruses: is there a relationship? Front Biosci (Elite Ed). (2014) 6:46–54. 10.2741/e689 24389140

[13] Kennedy JL Pham S Borish L. Rhinovirus and asthma exacerbations. Immunol Allergy Clin North Am. (2019) 39:335–44. 10.1016/j.iac.2019.03.003 31284924 PMC6625523

[14] Edwards MR Walton RP Jackson DJ Feleszko W Skevaki C Jartti T. EAACI anti-infectives in asthma and asthma exacerbations task force. The potential of anti-infectives and immunomodulators as therapies for asthma and asthma exacerbations. Allergy. (2018) 73:50–63. 10.1111/all.13257 28722755 PMC7159495

[15] Robinson RF Nahata MC. Respiratory syncytial virus (RSV) immune globulin and palivizumab for prevention of RSV infection. Am J Health Syst Pharm. (2000) 57:259–64. 10.1093/ajhp/57.3.259 10674778

[16] Wong SK Li A Lanctôt KL Paes B. Adherence and outcomes: a systematic review of palivizumab utilization. Expert Rev Respir Med. (2018) 12:27–42. 10.1080/17476348.2018.1401926 29130355

[17] Igde M Kabasakal H Ozturk O Karatekin G Aygun C. Palivizumab prophylaxis, respiratory syncytial virus and subsequent development of asthma. Minerva Pediatr. (2018) 70:252–9. 10.23736/S0026-4946.16.04368-1 29795072

[18] Mosquera RA De Jesus-Rojas W Stark JM Yadav A Jon CK Atkins CL. Role of prophylactic azithromycin to reduce airway inflammation and mortality in a RSV mouse infection model. Pediatr Pulmonol. (2018) 53:567–74. 10.1002/ppul.23956 29405608

[19] Cardinale F Lombardi E Rossi O Bagnasco D Bellocchi A Menzella F. Epithelial dysfunction, respiratory infections and asthma: the importance of immunomodulation. A focus on OM-85. Expert Rev Respir Med. (2020) 14:1019–26. 10.1080/17476348.2020.1793673 32635771

[20] Esposito S Jones MH Feleszko W Martell JAO Falup-Pecurariu O Geppe N. Prevention of new respiratory episodes in children with recurrent respiratory infections: an expert consensus statement. Microorganisms. (2020) 8:1810. 10.3390/microorganisms8111810 33213053 PMC7698530

[21] Dondi A Calamelli E Piccinno V Ricci G Corsini I Biagi C. Acute asthma in the pediatric emergency department: infections are the main triggers of exacerbations. Biomed Res Int. (2017) 2017:9687061. 10.1155/2017/9687061 29159184 PMC5660758

[22] Darrow LA Hess J Rogers CA Tolbert PE Klein M Sarnat SE. Ambient pollen concentrations and emergency department visits for asthma and wheeze. J Allergy Clin Immunol. (2012) 130:630–8.e4. 10.1016/j.jaci.2012.06.020 22840851 PMC3432157

[23] Charpin D Ramadour M Lavaud F Raherison C Caillaud D de Blay F. Climate and allergic sensitization to airborne allergens in the general population: data from the french six cities study. Int Arch Allergy Immunol. (2017) 172:236–41. 10.1159/000471511 28456804

[24] Ahluwalia SK Matsui EC. Indoor environmental interventions for furry pet allergens, pest allergens, and mold: looking to the future. J Allergy Clin Immunol Pract. (2018) 6:9–19. 10.1016/j.jaip.2017.10.009 29310769 PMC5763515

[25] Murakami Y Honjo S Odajima H Adachi Y Yoshida K Ohya Y. Exercise-induced wheezing among Japanese pre-school children and pupils. Allergol Int. (2014) 63:251–9. 10.2332/allergolint.13-OA-0644 24759555

[26] Kuti BP Kuti DK Teague WG. Determinants of severe exercise-induced bronchoconstriction in Nigerian children with asthma. Pediatr Pulmonol. (2020) 55(Suppl. 1):S51–S60. 10.1002/ppul.24609 31990143

[27] Minic PB Sovtic AD. Exercise intolerance and exercise-induced bronchoconstriction in children. Front Biosci (Elite Ed). (2017) 9:21–32. Published (2017). Jan 1. 10.2741/e782 27814586

[28] Tikkakoski AP Tikkakoski A Kivistö JE Huhtala H Sipilä K Karjalainen J. Association of air humidity with incidence of exercise-induced bronchoconstriction in children. Pediatr Pulmonol. (2019) 54:1830–36. 10.1002/ppul.24471 31393065

[29] Lang JE. The impact of exercise on asthma. Curr Opin Allergy Clin Immunol. (2019) 19:118–25. 10.1097/ACI.0000000000000510 30601152

[30] Weinstein AG. Crying-induced bronchospasm in childhood asthma. J Asthma. (1984) 21:161–5. 10.3109/02770908409077415 6735973

[31] Liangas G Morton JR Henry RL. Mirth-triggered asthma: is laughter really the best medicine? Pediatr Pulmonol. (2003) 36:107–12. 10.1002/ppul.10313 12833489

[32] Sandberg S Paton JY Ahola S McCann DC McGuinness D Hillary CR. The role of acute and chronic stress in asthma attacks in children. Lancet. (2000) 356:982–7. 10.1016/S0140-6736(00)02715-X 11041399

[33] Wood BL Lim J Miller BD Cheah PA Simmens S Stern T. Family emotional climate, depression, emotional triggering of asthma, and disease severity in pediatric asthma: examination of pathways of effect. J Pediatr Psychol. (2007) 32:542–51. 10.1093/jpepsy/jsl044 17124184

[34] Landeo-Gutierrez J Forno E Miller GE Celedón JC. Exposure to violence, psychosocial stress, and asthma. Am J Respir Crit Care Med. (2020) 201:917–22. 10.1164/rccm.201905-1073PP 31801032 PMC7159436

[35] Miller GE Chen E. Life stress and diminished expression of genes encoding glucocorticoid receptor and beta2-adrenergic receptor in children with asthma. Proc Natl Acad Sci USA. (2006) 103:5496–501. 10.1073/pnas.0506312103 16567656 PMC1414639

[36] Baldwin CM Bell IR O'Rourke MK. Odor sensitivity and respiratory complaint profiles in a community-based sample with asthma, hay fever, and chemical odor intolerance. Toxicol Ind Health. (1999) 15:403–9. 10.1177/074823379901500314 10416292

[37] Elberling J Linneberg A Dirksen A Johansen JD Frølund L Madsen F. Mucosal symptoms elicited by fragrance products in a population-based sample in relation to atopy and bronchial hyper-reactivity. Clin Exp Allergy. (2005) 35:75–81. 10.1111/j.1365-2222.2005.02138.x 15649270

[38] Jaén C Dalton P. Asthma and odors: the role of risk perception in asthma exacerbation. J Psychosom Res. (2014) 77:302–8. 10.1016/j.jpsychores.2014.07.002 25280827 PMC4734637

[39] Mireku N Wang Y Ager J Reddy RC Baptist AP. Changes in weather and the effects on pediatric asthma exacerbations. Ann Allergy Asthma Immunol. (2009) 103:220–4. 10.1016/S1081-1206(10)60185-8 19788019

[40] Schinasi LH Kenyon CC Moore K Melly S Zhao Y Hubbard R. Heavy precipitation and asthma exacerbation risk among children: a case-crossover study using electronic health records linked with geospatial data. Environ Res. (2020) 188:109714. 10.1016/j.envres.2020.109714 32559685

[41] Hu Y Cheng J Jiang F Liu S Li S Tan J. Season-stratified effects of meteorological factors on childhood asthma in Shanghai, China. Environ Res. (2020) 191:110115. 10.1016/j.envres.2020.110115 32846175

[42] Silverman RA Ito K. Age-related association of fine particles and ozone with severe acute asthma in New York City. J Allergy Clin Immunol. (2010) 125:367–373.e5. 10.1016/j.jaci.2009.10.061 20159246

[43] Zhao S Yu Y Yin D He J Liu N Qu J. Annual and diurnal variations of gaseous and particulate pollutants in 31 provincial capital cities based on *in situ* air quality monitoring data from China national environmental monitoring center. Environ Int. (2016) 86:92–106. 10.1016/j.envint.2015.11.003 26562560

[44] Lu P Zhang Y Lin J Xia G Zhang W Knibbs LD. Multi-city study on air pollution and hospital outpatient visits for asthma in China. Environ Pollut. (2020) 257:113638. 10.1016/j.envpol.2019.113638 31812526

[45] Strickland MJ Darrow LA Klein M Flanders WD Sarnat JA Waller LA. Short-term associations between ambient air pollutants and pediatric asthma emergency department visits. Am J Respir Crit Care Med. (2010) 182:307–16. 10.1164/rccm.200908-1201OC 20378732 PMC2921597

[46] Houdouin V Dubus JC. What is the impact of outdoor pollution on children's asthma? Arch Pediatr. (2019) 26:487–91. 10.1016/j.arcped.2019.10.007 31685409

[47] Murray AB Morrison BJ. Passive smoking by asthmatics: its greater effect on boys than on girls and on older than on younger children. Pediatrics. (1989) 84:451–9. 10.1203/00006450-198909000-00118 2771548

[48] Dong GH Cao Y Ding HL Ma YN Jin J Zhao YD. Effects of environmental tobacco smoke on respiratory health of boys and girls from kindergarten: results from 15 districts of northern China. Indoor Air. (2007) 17:475–83. 10.1111/j.1600-0668.2007.00495.x 18045272

[49] Gergen PJ Fowler JA Maurer KR Davis WW Overpeck MD. The burden of environmental tobacco smoke exposure on the respiratory health of children 2 months through 5 years of age in the United States: Third National Health and Nutrition Examination Survey, 1988 to (1994). Pediatrics. (1998) 101:E8. 10.1542/peds.101.2.e8 9445518

[50] Wang Z May SM Charoenlap S Pyle R Ott NL Mohammed K. Effects of secondhand smoke exposure on asthma morbidity and health care utilization in children: a systematic review and meta-analysis. Ann Allergy Asthma Immunol. (2015) 115:396–401.e2. 10.1016/j.anai.2015.08.005 26411971

[51] Neophytou AM Oh SS White MJ Mak ACY Hu D Huntsman S. Secondhand smoke exposure and asthma outcomes among African-American and Latino children with asthma. Thorax. (2018) 73:1041–8. 10.1136/thoraxjnl-2017-211383 29899038 PMC6225993

